# Identification and Localization of *Myxococcus xanthus* Porins and Lipoproteins

**DOI:** 10.1371/journal.pone.0027475

**Published:** 2011-11-22

**Authors:** Swapna Bhat, Xiang Zhu, Ricky P. Patel, Ron Orlando, Lawrence J. Shimkets

**Affiliations:** 1 Department of Microbiology, University of Georgia, Athens, Georgia, United States of America; 2 Complex Carbohydrate Research Center, University of Georgia, Athens, Georgia, United States of America; East Carolina University School of Medicine, United States of America

## Abstract

*Myxococcus xanthus* DK1622 contains inner (IM) and outer membranes (OM) separated by a peptidoglycan layer. Integral membrane, β-barrel proteins are found exclusively in the OM where they form pores allowing the passage of nutrients, waste products and signals. One porin, Oar, is required for intercellular communication of the C-signal. An *oar* mutant produces CsgA but is unable to ripple or stimulate *csgA* mutants to develop suggesting that it is the channel for C-signaling. Six prediction programs were evaluated for their ability to identify β-barrel proteins. No program was reliable unless the predicted proteins were first parsed using Signal P, Lipo P and TMHMM, after which TMBETA-SVM and TMBETADISC-RBF identified β-barrel proteins most accurately. 228 β-barrel proteins were predicted from among 7331 protein coding regions, representing 3.1% of total genes. Sucrose density gradients were used to separate vegetative cell IM and OM fractions, and LC-MS/MS of OM proteins identified 54 β-barrel proteins. Another class of membrane proteins, the lipoproteins, are anchored in the membrane via a lipid moiety at the N-terminus. 44 OM proteins identified by LC-MS/MS were predicted lipoproteins. Lipoproteins are distributed between the IM, OM and ECM according to an N-terminal sorting sequence that varies among species. Sequence analysis revealed conservation of alanine at the +7 position of mature ECM lipoproteins, lysine at the +2 position of IM lipoproteins, and no noticable conservation within the OM lipoproteins. Site directed mutagenesis and immuno transmission electron microscopy showed that alanine at the +7 position is essential for sorting of the lipoprotein FibA into the ECM. FibA appears at normal levels in the ECM even when a +2 lysine is added to the signal sequence. These results suggest that ECM proteins have a unique method of secretion. It is now possible to target lipoproteins to specific IM, OM and ECM locations by manipulating the amino acid sequence near the +1 cysteine processing site.

## Introduction

The life cycle of *Myxococcus xanthus* involves a vegetative stage, in which cells feed on bacteria and organic detritus, and a developmental stage in which thousands of cells aggregate to form a multicellular fruiting body containing spores. Fruiting body development involves intercellular communication with at least six extracellular signals [1]. However, the receptors and sensory pathways of these signaling pathways are largely unknown. Identification of outer membrane (OM) proteins in *M. xanthus* may reveal components of these signaling pathways that are used to export or import signals.

The OM acts as a selective barrier that allows the passage of nutrients, water and chemical signals through pores formed by porin proteins. In porins, antiparallel β-strands are arranged to form a cylindrical β-barrel structure lined with hydrophilic residues that create a water-filled channel [2]. Some porins allow passive diffusion of small solutes with molecular weights up to 600 Da [3]. Active diffusion of specific nutrients through porins is carried out by TonB systems, which utilize energy provided by the inner membrane (IM) to mediate solute passage through the OM [4]. Some porins allow passage of specific substrates, such as fatty acids in the case of FadL [5]. Porins are synthesized as precursors with an N-terminal signal sequence that aids transport across the IM via the general secretory (Sec) pathway [6]. The signal sequences are hydrolyzed by signal peptidases present in the IM. Chaperones in the periplasm facilitate protein folding and insertion into the OM using the Omp85 machinery [7].

Databases such as Pfam can help identify OM proteins, but only if the protein contains a domain with appreciable identity to a domain of known function [8]. Unfortunately, most bacterial genomes contain hypothetical proteins that are not represented in the Pfam database. For example, the *M. xanthus* genome encodes 40% hypothetical proteins [9]. Thus, bioinformatic programs that can predict the OM protein β-barrel structure would be useful since this structure is unique to porins [10].

The IM and OM also contains lipoproteins that are anchored by a lipid-modified N-terminal cysteine residue. Lipoproteins are transported as precursors via the Sec pathway to the IM where they are processed at the conserved N-terminal lipobox. The lipobox consists of four amino acids (L_−3_-[A/S/T]_−2_-[G/A]_−1_-C_+1_) around the signal peptide cleavage site with the +1 cysteine serving as the site of covalent modification [11]. Lipoprotein maturation involves attachment of a diacylglycerol group to the +1 cysteine sulfhydryl group via a thioester linkage, cleavage of the signal peptide, and acylation of the +1 α-amino group. The lipid moieties anchor the N-terminus of the proteins in lipid bilayers.

In *Escherichia coli*, localization of lipoproteins to the IM requires aspartate at the +2 position of the mature lipoprotein [12]. Lipoproteins lacking this sorting signal are transported to the OM via the Lol pathway, which is an ABC transport system located in the IM [13]. The signal sequences directing Lol avoidance differ among bacteria. In *Pseudomonas aeruginosa*, lysine and serine at positions +3 and +4 lead to IM retention of lipoproteins [14]. Some bacteria secrete lipoproteins and may possess novel mechanisms to sort lipoproteins to the external environment [15]. *M. xanthus* secretes at least 11 lipoproteins to the extracellular matrix (ECM) whose mechanism of targeting is unknown [16].

In this paper we identified OM proteins using bioinformatic and proteomic tools. Two prediction programs TMBETA-SVM and TMBETADISC-RBF identified 228 β-barrel OM proteins in the genome, of which 54 were detected in DK1622 vegetative cells by LC-MS/MS. We show that one of these proteins, Oar, is essential for C-signal transmission during fruiting body development. Lipoprotein sorting into IM, OM, and extracellular compartments was also examined. Alanine at the +7 position mediates ECM localization, even when a signal for IM localization is also present, suggesting that there are at least two lipoprotein secretion pathways.

## Results

The first goal of this study was to identify *M. xanthus* OM porins and lipoproteins using bioinformatic and proteomic approaches, then examine one porin, Oar, for a role in fruiting body development. The second goal of this study was to identify the trafficking signals for IM, OM and ECM lipoproteins. Site directed mutagenesis was then used to identify the ECM trafficking signal for the major ECM lipoprotein FibA.

### β-barrel prediction in *M. xanthus* proteome

**Figure 1:**
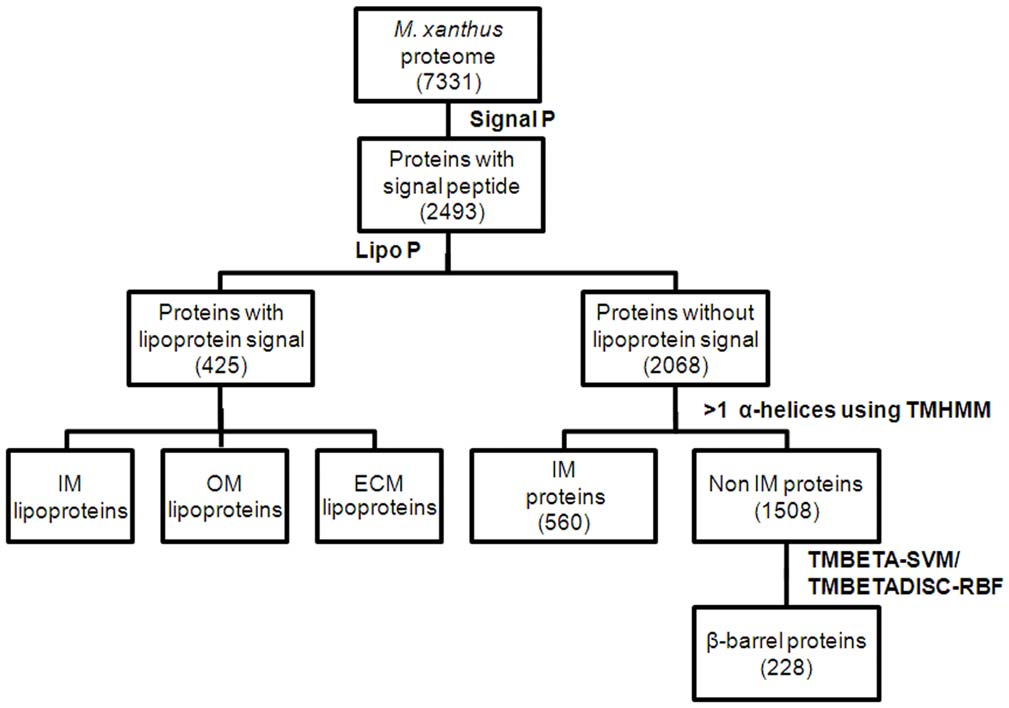
Scheme to identify OM proteins utilizing bioinformatic programs. The Signal P program identified 2493 signal peptide containing proteins among the putative 7331 member *M. xanthus* proteome. Of these 425 lipoproteins were identified using Lipo P. Of the 2068 proteins without a lipoprotein signal 560 were integral IM proteins identified using TMHMM. The non-IM proteins include periplasmic proteins, secreted proteins and OM proteins. Finally, integral OM proteins containing a β-barrel domain were identified using TMBETA-SVM plus TMBETADISC-RBF.

Integral OM proteins are synthesized with an N-terminal signal sequence. The signal sequence enables transport of proteins across the IM by the Sec system, and can be predicted using the program Signal P [17]. The *M. xanthus* proteome was examined for candidates with a signal peptide, which generated 2493/7331 candidates (Figure 1). In the next step, predicted signal peptide containing proteins were classified as lipoproteins or non-lipoproteins using the Lipo P program [13]. 425 out of 2493 signal peptide-containing proteins were predicted to be lipoproteins (Figure 1). The non-lipoproteins were further segregated into IM and non-IM proteins. Integral IM proteins have transmembrane alpha helices that are rich in hydrophobic amino acids. While some integral membrane proteins have only a single transmembrane domain, we felt that parsing out proteins with a single predicted transmembrane domain was a bit risky. Therefore, proteins with at least two putative transmembrane helices using the TMHMM program were classified as IM proteins [13]. 560/2068 (27%) proteins with putative signal peptides were identified as IM proteins.

The remaining 1508 signal peptide containing proteins, comprising periplasmic, secreted, and OM proteins, were subjected to β-barrel prediction programs to identify integral OM proteins. Six prediction programs were evaluated, TMBETA-NET, PRED-TMββ, TMBETA-SVM, TMBETADISC-RBF, BOMP, and TMB-HUNT, using 40 *M. xanthus* protein sequences with predicted β-barrel domains obtained from the Pfam database (Table 1). These proteins are homologous to well-studied OM proteins from other organisms as demonstrated by BLASTP. The 40 proteins represent 10 protein families including TonB dependent receptors, Omp85 and OmpH, secretins, efflux proteins, and organic solvent tolerance proteins [18], [19], [20], [21]. Except for BOMP (10%) and TMB-HUNT (63%) all prediction programs identified >77% of the 40 OM proteins (Table 1). PRED-TMββ identified the most β-barrel proteins (38/40) (95%). TMBETADISC-RBF, TMBETA-SVM and TMBETA-NET identified 34/40 (85%), 33/40 (83%) and 31/40 (78%) β-barrel proteins, respectively.

**Table 1:** *M. xanthus* β-barrel domain proteins obtained from the Pfam database.

MXAN Number	Function	1^a^	2^b^	3^c^	4^d^	5^e^	6^f^
AN0272	TonB dependent receptor	+	+	+	+	+	+
MXAN0518	TonB dependent receptor	+	+	+	−	−	−
MXAN0562	Phosphate selective porin (PhoE)	+	+	+	+	−	+
MXAN0990	Outer membrane efflux protein	+	−	+	−	−	−
MXAN1316	TonB dependent receptor	+	+	+	+	+	−
MXAN1450	TonB dependent receptor (Oar)	+	+	+	+	+	−
MXAN2514	Secretin (GspD)	+	+	+	+	−	−
MXAN2708	Organic solvent tolerance protein (OstA)	+	+	+	+	+	−
MXAN3106	Secretin (GspE)	+	+	+	+	+	−
MXAN3431	Outer membrane efflux protein	+	+	+	−	−	−
MXAN3883	Fimbrial usher protein (FUP)	+	+	+	+	+	−
MXAN3905	Outer membrane efflux protein	+	+	+	+	+	−
MXAN4176	Outer membrane efflux protein	+	+	+	+	+	−
MXAN4198	Outer membrane efflux protein	+	+	+	+	+	−
MXAN4559	TonB dependent receptor	+	+	+	+	+	−
MXAN4727	Structural protein (OmpH)	+	+	−	−	+	−
MXAN4728	Omp85 protein	+	+	+	−	−	−
MXAN4746	TonB dependent receptor	+	+	+	+	+	−
MXAN4772	OmpA protein	−	+	−	−	+	−
MXAN5023	TonB dependent receptor	+	+	+	+	−	−
MXAN5030	Outer membrane efflux transporter	+	+	+	+	+	−
MXAN5042	OmpA protein	+	−	−	−	−	−
MXAN5069	Aquaporin Z (ApqZ)	+	−	−	−	+	−
MXAN5772	Secretin (PilQ)	+	+	+	+	+	−
MXAN5956	Major intrinsic protein	+	−	+	−	−	−
MXAN6044	TonB dependent receptor	+	+	+	+	+	−
MXAN6176	Outer membrane efflux protein	+	+	+	+	+	−
MXAN6246	OmpA	+	−	−	−	−	−
MXAN6487	Outer membrane efflux protein	+	+	+	+	+	−
MXAN6547	TonB dependent receptor	+	+	+	+	−	−
MXAN6579	TonB dependent receptor	+	+	+	+	+	−
MXAN6716	TonB dependent receptor	+	+	+	+	−	−
MXAN6845	TonB dependent receptor	+	+	+	+	+	−
MXAN6911	TonB dependent receptor	+	+	+	+	+	+
MXAN7037	OmpA	+	+	+	+	+	−
MXAN7040	Fatty acid transport (FadL)	+	+	+	+	+	+
MXAN7238	Outer membrane efflux protein	+	+	−	+	+	−
MXAN7331	TonB dependent receptor	+	+	+	+	−	−
MXAN7397	OmpA	−	−	−	−	+	−
MXAN7436	Outer membrane efflux protein	+	+	+	+	−	−

Programs that correctly predicted a β-barrel protein are indicated by a positive sign (+) while failing to do so is indicated by a negative sign (−).

a PRED-TMββ.

b TMBETADISC-RBF.

c TMBETA-SVM.

d TMBETA-NET.

e TMB-HUNT.

f BOMP.

TMBETA-NET, TMBETA-SVM, TMBETADISC-RBF and PRED-TMββ were tested on 21 *M. xanthus* IM, periplasmic, and ECM proteins to eliminate programs that generate false positives (Table 2). The IM and periplasmic proteins were obtained from the Pfam database while the ECM proteins were previously identified from proteomic studies [16]. PRED-TMββ produced 11/20 (55%) false positives, TMBETA-NET and TMBETADISC-RBF generated 2/20 (9.5%) false positives, and TMBETA-SVM produced no false positives.

**Table 2:** *M. xanthus* IM, periplasmic and ECM proteins^1^.

MXAN Number	Function	Predicted localization	1^a^	2^b^	3^c^	4^d^
MXAN0468	peptidylprolyl cis-trans isomerase	Periplasm	+	−	−	−
MXAN0977	di-haem cytochrome-c peroxidase	Periplasm	−	−	−	−
MXAN1066	PTS system, IIA component	Periplasm	+	−	−	−
MXAN1389	alkaline phosphatase	Periplasm	+	−	−	−
MXAN2832	permease	Periplasm	−	−	−	−
MXAN2951	ABC transporter, periplasmic substrate binding protein	Periplasm	−	−	−	−
MXAN3420	multicopper oxidase (CumA)	Periplasm	−	−	−	−
MXAN0274	biopolymer transport protein, ExbD/TolR family	IM	−	−	−	−
MXAN0559	ABC transporter,ATP-binding protein (Mac1)	IM	+	−	−	−
MXAN2505	general secretory pathway protein K (GspK)	IM	−	−	−	+
MXAN2570	acetate–CoA ligase	IM	−	−	−	−
MXAN3182	Serine threonine kinase	IM	+	−	−	−
MXAN4829	isoquinoline 1-oxidoreductase, beta subunit (IorB)	IM	+	−	−	−
MXAN5123	sensor histidine kinase MrpA (MrpA)	IM	+	+	−	−
MXAN0075	amidohydrolase	ECM	−	−	−	−
MXAN1424	unknown	ECM	+	+	−	−
MXAN1493	unknown	ECM	+	−	−	−
MXAN2375	unknown	ECM	−	−	−	−
MXAN3885	Spore coat U	ECM	+	−	−	+
MXAN5686	unknown	ECM	+	−	−	−

1 IM and periplasmic proteins were obtained from the Pfam database while ECM proteins were previously identified by Curtis et al [16].

a PRED-TMββ.

b TMBETADISC-RBF.

c TMBETA-SVM.

d TMBETA-NET.

In the absence of a stand-alone program for TMBETA-NET, β-barrel proteins were identified using TMBETA-SVM and TMBETADISC-RBF, which predicted 240/1508 and 414/1508 proteins respectively. 228 proteins were identified by both programs equivalent to ∼3.1% of the genome (Table S1). Analyses of several Gram-negative bacteria suggests that 2–3% of the genome encodes porins [22].

### Identification of OM proteins in *M. Xanthus*

The OM fraction was purified, then subjected to LC-MS/MS [23]. Using the bioinformatic scheme shown in figure 1, 54 β-barrel proteins (Table 3) were identified along with 44 lipoproteins (Table 4). The bioinformatic scheme enabled the identification of cytoplasmic, IM, and periplasmic contaminants in the OM preparation (Table S2).

**Table 3:** OM β-barrel proteins identified by LC-MS/MS.

MXAN Number^1^	Function	No. of peptides
MXAN0219^a^	Hypothetical protein	1
MXAN0518^a^	TonB-dependent receptor	1
MXAN0659^b^	Putative lipoprotein	5
MXAN0662^b^	Hypothetical protein	3
MXAN0751^b^	Conserved domain protein	4
MXAN0855^b^	Putative chemotaxis MotB protein	5
MXAN0924^a^	Hypothetical protein	2
MXAN1426^b^	Hypothetical protein	2
MXAN1450^a^	TonB-dependent receptor (Oar)	40
MXAN1689	Conserved hypothetical protein	1
MXAN2417^b^	Conserved hypothetical protein	1
MXAN2462^b^	Hypothetical protein	1
MXAN2514^a^	General secretion pathway protein D	4
MXAN2536	Putative long-chain-fatty-acid-CoA ligase	6
MXAN2659^a^	Hypothetical protein	17
MXAN2906^b^	Penicillin acylase family protein	8
MXAN3106^a^	Bacterial membrane secretin (secretin) family	10
MXAN3160^b^	Peptidase, M13 (neprilysin) family	22
MXAN3774^b^	Conserved Hypothetical protein	11
MXAN3780	Patatin-like phospholipase family protein	3
MXAN3953^b^	Hypothetical protein	2
MXAN4085	Peptidylprolyl cis-trans isomerase, FKBP-type	1
MXAN4293^b^	Hypothetical protein	4
MXAN4295	Patatin-like phospholipase family protein	6
MXAN4365	Outer membrane receptor family	1
MXAN4652	Putative Flp pilus assembly protein CpaB	1
MXAN4728^a^	OMP85 family protein	4
MXAN4746^a^	TonB-dependent receptor	5
MXAN5023^a^	TonB dependent receptor	5
MXAN5152^b^	OmpA family protein	3
MXAN5194	OmpA domain protein	2
MXAN5453^b^	Hypothetical protein	6
MXAN5685^b^	Hypothetical protein	2
MXAN5743^b^	Hypothetical protein	11
MXAN5756^b^	TolB protein	2
MXAN5931^a^	Hypothetical protein	7
MXAN6079^b^	Putative molybdopterin oxidoreductase, iron-sulfur binding subunit	15
MXAN6090^b^	Hypothetical protein	10
MXAN6196^a^	Hypothetical protein	3
MXAN6487^a^	Outer membrane efflux protein domain protein	11
MXAN6521^b^	Putative lipoprotein	1
MXAN6829	Hypothetical protein	4
MXAN6891^b^	Hypothetical protein	2
MXAN6911^a^	TonB-dependent receptor	8
MXAN7037	Putative chemotaxis MotB protein	1
MXAN7039^b^	Putative lipoprotein	34
MXAN7040^a^	FadL	13
MXAN7104^b^	M3 (thimet oligopeptidase) family peptidase	10
MXAN7112^b^	Conserved Hypothetical protein	3
MXAN7196^b^	Hypothetical protein	1
MXAN7203^a^	Putative 28 kDa outer membrane protein	5
MXAN7407^a^	Hypothetical protein	6
MXAN7436^a^	Outer membrane efflux protein	4
MXAN7513	Hypothetical protein	1

1 All proteins labeled with an ‘a’ or ‘b’, were identified in both this study and the Kahnt et al. study using proteomic approaches. Proteins identified by proteomics and classified as β-barrel proteins in the Kahnt et al. study, are identified with an ‘a’ superscript. Proteins identified by proteomics in the Kahnt et al. study and likely misclassified by them, are denoted with a ‘b’. Proteins with no superscript are β-barrel proteins unique to this study.

**Table 4:** OM lipoproteins identified by LC-MS/MS.

MXAN Number	Function	No. of peptides
MXAN0283	Putative lipoprotein	1
MXAN0522	Putative lipoprotein	2
MXAN0533	NAD dependent epimerase/dehydratase family	1
MXAN0662	Hypothetical protein	3
MXAN0751	Conserved domain protein	4
MXAN0934	Protease DO family protein	14
MXAN1063	Putative lipoprotein	1
MXAN1162	Putative lipoprotein	5
MXAN1176	Peptidylprolyl cis-trans isomerase, cyclophilin-type	1
MXAN1342	Putative lipoprotein	1
MXAN1451	Putative lipoprotein MlpA	4
MXAN1623	peptidase, M16 (pitrilysin) family	8
MXAN1689	Conserved hypothetical protein	1
MXAN2091	Peptidase, M16 (pitrilysin) family	3
MXAN2286	Peptidyl-dipeptidase Dcp	4
MXAN2417	Conserved hypothetical protein	1
MXAN2470	5′-nucleotidase family protein	1
MXAN2660	Putative lipoprotein	6
MXAN2968	Efflux transporter, RND family, MFP subunit	4
MXAN3060	Adventurous gliding motility protein CglB	4
MXAN3084	Social gliding motility protein Tgl	2
MXAN3103	Putative lipoprotein	3
MXAN3440	Peptidase, M13 (neprilysin) family	4
MXAN3581	Peptidyl-dipeptidase A	4
MXAN4641	Hypothetical protein	1
MXAN4747	Putative lipoprotein	1
MXAN4900	Putative lipoprotein	15
MXAN4966	Putative lipoprotein	10
MXAN5331	Putative lipoprotein	2
MXAN5361	Putative 5′-nucleotidase	1
MXAN5390	Putative lipoprotein	1
MXAN5684	Putative lipoprotein	5
MXAN5933	Peptidase, M48 (Ste24 endopeptidase) family	5
MXAN6381	Hypothetical protein	1
MXAN6521	Putative lipoprotein	1
MXAN6660	Hypothetical protein	3
MXAN6720	Putative lipoprotein	2
MXAN6978	Putative lipoprotein	2
MXAN6985	Hypothetical protein	1
MXAN7108	Putative lipoprotein	1
MXAN7110	Peptidyl-prolyl cis-trans isomerase, FKBP-type	8
MXAN7220	Putative lipoprotein	1
MXAN7333	Putative lipoprotein	4
MXAN7438	Putative cobalt-zinc-cadmium resistance protein	3

### Oar is required for C-signaling

**Figure 2:**
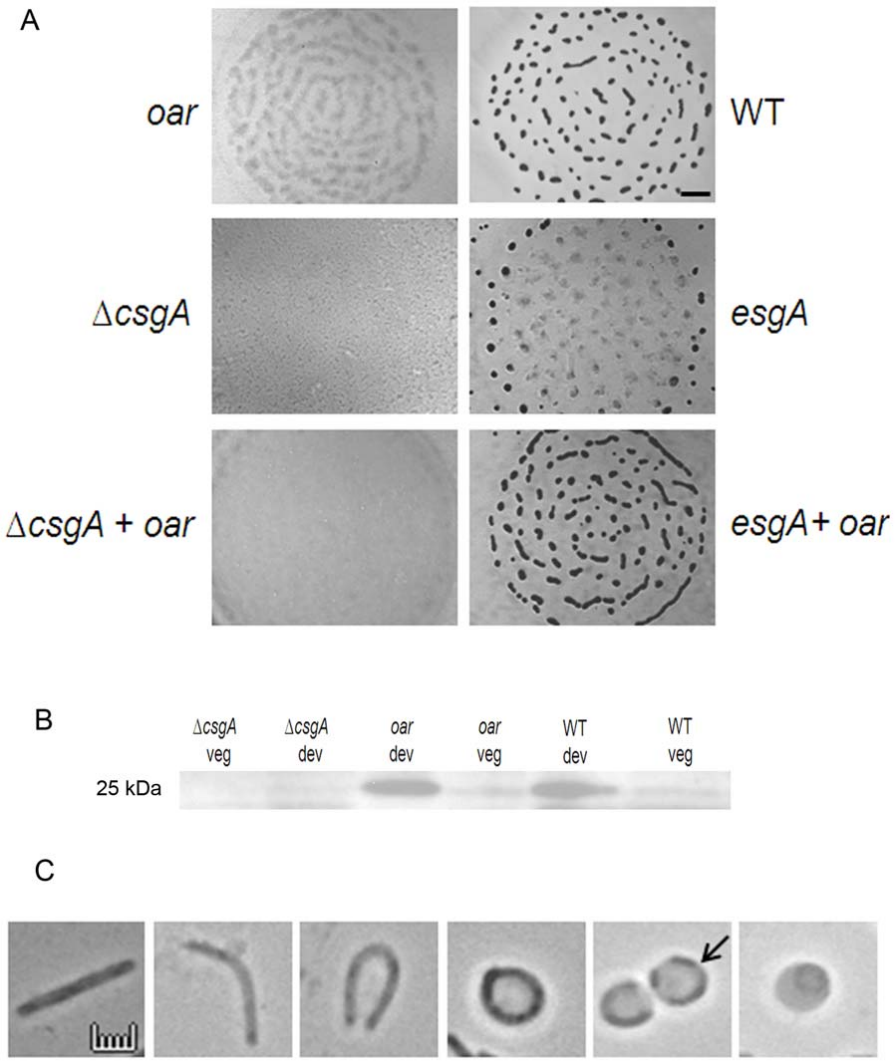
Role of *oar* in cell signaling. (A) Extracellular complementation of *oar* (LS2453) cells with Δ*csgA* (LS2441) and *esg* (JD300). WT (DK1622) cells were used as a control. Bar is 1 mm (B) Western blot analysis of vegetative cells and 24 h developing cells using anti-CsgA primary antibody. (C) Morphology of *oar* cells during development. The first panel represents 24 h developing WT cells while the subsequent panels represent various *oar* cell shapes as they ultimately transform into spheroplast (extreme right panel). Bar is 1 µm.

Porins form hydrophilic channels through which extracellular signals may pass. *oar* mutants exhibit delayed aggregation and are unable to sporulate [24]. Oar appears to be a TonB dependent receptor. To determine whether an essential developmental signal passes through the Oar pore, *oar* cells were mixed pair wise with mutants unable to produce each of the essential developmental signals A, B, C, D, E and S [1]. If the *oar* mutant is proficient in producing an extracellular signal, it would be expected to rescue development of a mutant unable to produce such a signal. *oar* cells were mixed in 1∶1 ratio with mutants from each signal-producing class, and rescued development of all but *csgA* mutants (Figure 2A).

The *csgA* gene is required for the production of the C-signal [25]. Δ*csgA* cells can be rescued for development by mixing with *csgA* ^+^ cells even if those cells are also developmentally defective [26]. 1∶1 mixtures containing *oar* and Δ*csgA* cells failed to undergo fruiting body development suggesting that *oar* cells are unable to provide C-signal to Δ*csgA* cells. In contrast, the *esg* gene product is required for the synthesis of a branched chain fatty acid required for production of the E-signal [27]. *esg* mutations do not completely eliminate synthesis of branched chain fatty acids because there is a second pathway that can also produce them, albeit at smaller concentrations, causing some fruiting body development and sporulation as observed in Figure 2A. When *oar* cells were mixed with *esg* mutant cells, fruiting body development and sporulation was restored to wild type levels (Figure 2A).

*oar* cells produce CsgA at levels comparable to WT cells (Figure 2B), suggesting that *oar* has difficulty transmitting the C-signal. Another property suggestive of a defect in C-signaling is the inability to ripple (data not shown). During early development cells move in traveling waves known as ripples. Rippling requires C-signaling to regulate the cellular reversal period [28], [29]. The absence of both rippling and extracellular complementation of *csgA* mutants suggests a defect in C-signal transmission. However, the *oar* phenotype does not entirely phenocopy *csgA*. When the *oar* mutant was mixed 1∶1 with WT cells, 99% of the spores that germinated were of WT origin suggesting that the *oar* sporulation defect cannot be bypassed with an extracellular signal. *csgA* mutants, in contrast, typically form 30–50% of the spores when mixed with WT.

Examination of *oar* cells revealed a striking and novel defect. While 100% of 24 h developing WT cells show rod-shaped morphology, 62% of the *oar* cells were bent into horseshoe shapes that eventually circularize. The outer membrane appears to pull away from the rest of the cell to form spheroplasts (Figure 2c). These defects were not observed in *oar* vegetative cells (data not shown). In a *csgA oar* double mutant, only 8% of cells had *oar*-like, morphology. These results suggested that C-signal accumulation causes deformation of the cell envelop with lethal consequences.

### ECM and IM lipoproteins utilize different sorting signals in *M. xanthus*

**Figure 3:**
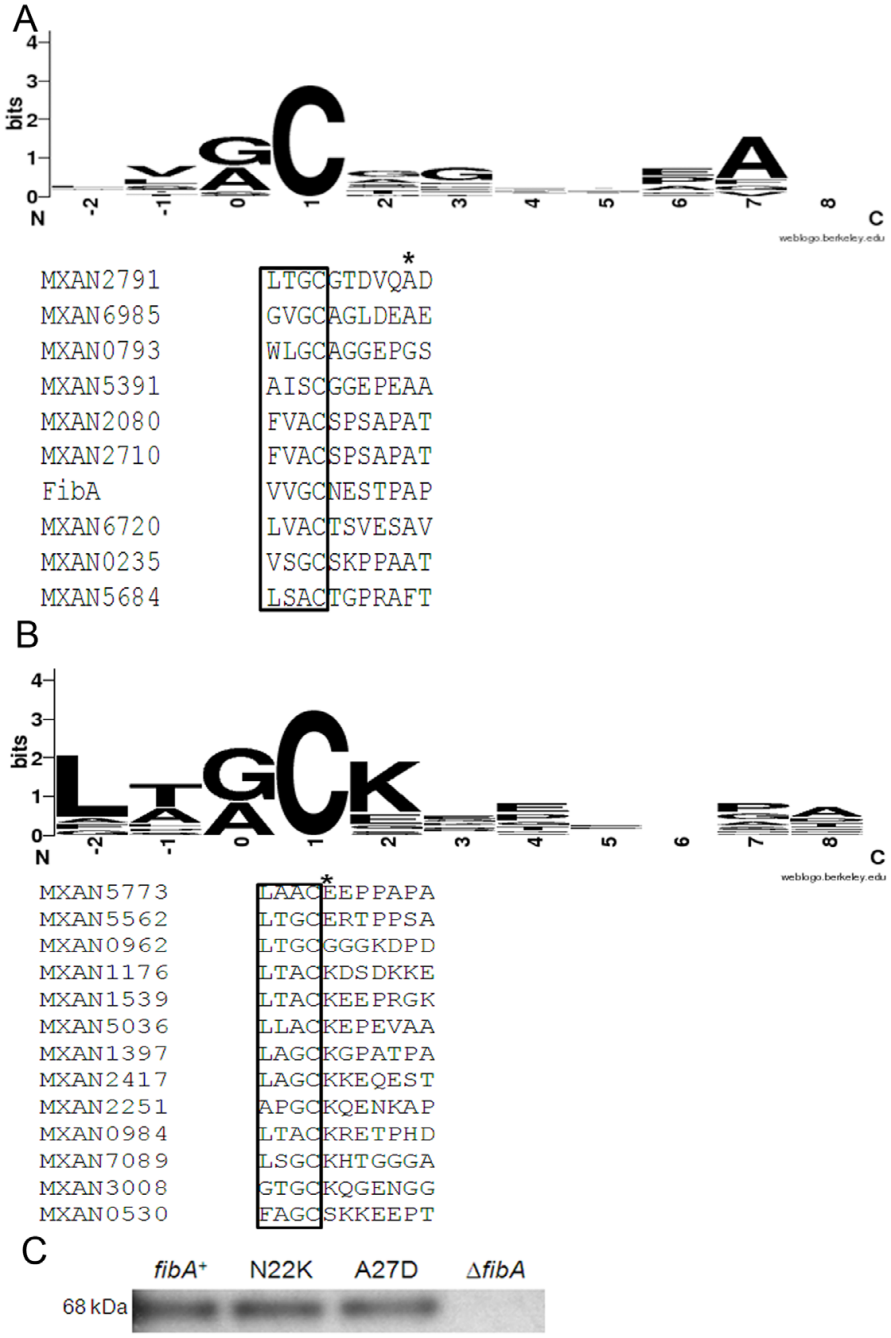
Bioinformatic analysis of first eight amino acids of N-terminus of *M. xanthus* lipoproteins. (A) Multiple sequence alignment of ECM proteins using WebLogo. The lipobox (highlighted by a box made of dashed lines) and the following seven amino acids of the N-terminal region of mature lipoproteins were aligned using WebLogo. Seven ECM lipoproteins have alanine at the 7^th^ position (highlighted by a solid box). (B) 8/12 predicted IM lipoproteins have lysine at the 2^nd^ position (highlighted by a solid box). (C) Western blot analysis of 18 h developing cells using Mab2105 primary antibody. Strains used include LS2760 (WT FibA), LS2208 (Δ*fibA*), LS2761 (N22K FibA), LS2764 (A27D FibA).

In other Proteobacteria, lipoproteins are sorted into the IM and OM using amino acid residues near the lipid modification site. Since *E. coli* does not secrete lipoproteins, the mechansim by which they make their way to the ECM are largely unknown. To investigate lipoprotein targeting to the ECM, the lipobox and the first eight amino acids of the mature ECM and IM lipoproteins were examined by multiple sequence alignment using WebLogo [30]. 7/10 ECM proteins, including by far the most abundant ECM protein FibA, possess alanine at the +7 position (Figure 3A). No amino acid conservation was observed in OM lipoproteins identified by LC-MS/MS (data not shown). This result suggests that +7 alanine may have a role in targeting lipoproteins to the ECM.

Because the mechanism of inner membrane targeting is also unknown in *M. xanthus*, and highly variable among the Proteobacteria, 12 putative IM lipoproteins were identified by LC-MS/MS from purified inner membranes (Table 5). 8/12 IM lipoproteins have lysine at the second position suggesting that lysine at the +2 position may be be essential for inner membrane targeting, as it is in *E. coli* (Figure 3B). The signal sequence of MXAN1176, a lipoprotein with lysine at the +2 position, localized mCherry in the IM [31].

**Table 5:** Putative lipoproteins identified by LC-MS/MS from IM fraction.

MXAN	Function	No. of peptides
MXAN 0530	Putative lipoprotein	1
MXAN 0962	Putative lipoprotein	2
MXAN 0984	Heavy metal efflux transporter, RND family, MFP subunit	3
MXAN 1176	Peptidylprolyl cis-trans isomerase, cyclophilin-type	5
MXAN 1397	PBS lyase HEAT-like repeat protein	3
MXAN 1539	Putative lipoprotein	6
MXAN 2417	Conserved hypothetical protein	8
MXAN 3008	Adventurous gliding motility protein AglU	5
MXAN 5036	Conserved domain protein	4
MXAN 5562	Putative lipoprotein	3
MXAN 5773	Putative lipoprotein	3
MXAN 7089	Putative lipoprotein	1

Site directed mutagenesis of *fibA* was carried out to determine whether alanine at the +7 position is essential for ECM localization. Since no amino acid conservation was observed in the N-terminus of OM lipoproteins, alanine (GCC) was changed to aspartate (GAC) at the 27^th^ position (+7 in mature FibA) as this substitution involved minimal nucleotide modification. The modified *fibA* gene was expressed in plasmid pZJY156 under control of the constitutive *pilA* promoter then introduced into Δ*fibA* strain LS2208. As a positive control, WT *fibA*, was introduced into LS2208 with the same vector system. Both strains produced comparable amounts of FibA as revealed by Western analysis of whole cells (Figure 3C). Immuno transmission electron microscopy was carried out to quantify FibA localization in the ECM. Cells were allowed to form biofilms on formvar coated nickel grids in submerged culture [32]. FibA secretion was then induced by incubating the grids in cohesion buffer. The cells were probed with anti-FibA (Mab2105) followed by anti-mouse antibody conjugated to 10 nm gold particles, and then examined by transmission electron microscopy. Gold particles on or around 10–12 cells were enumerated.

**Figure 4:**
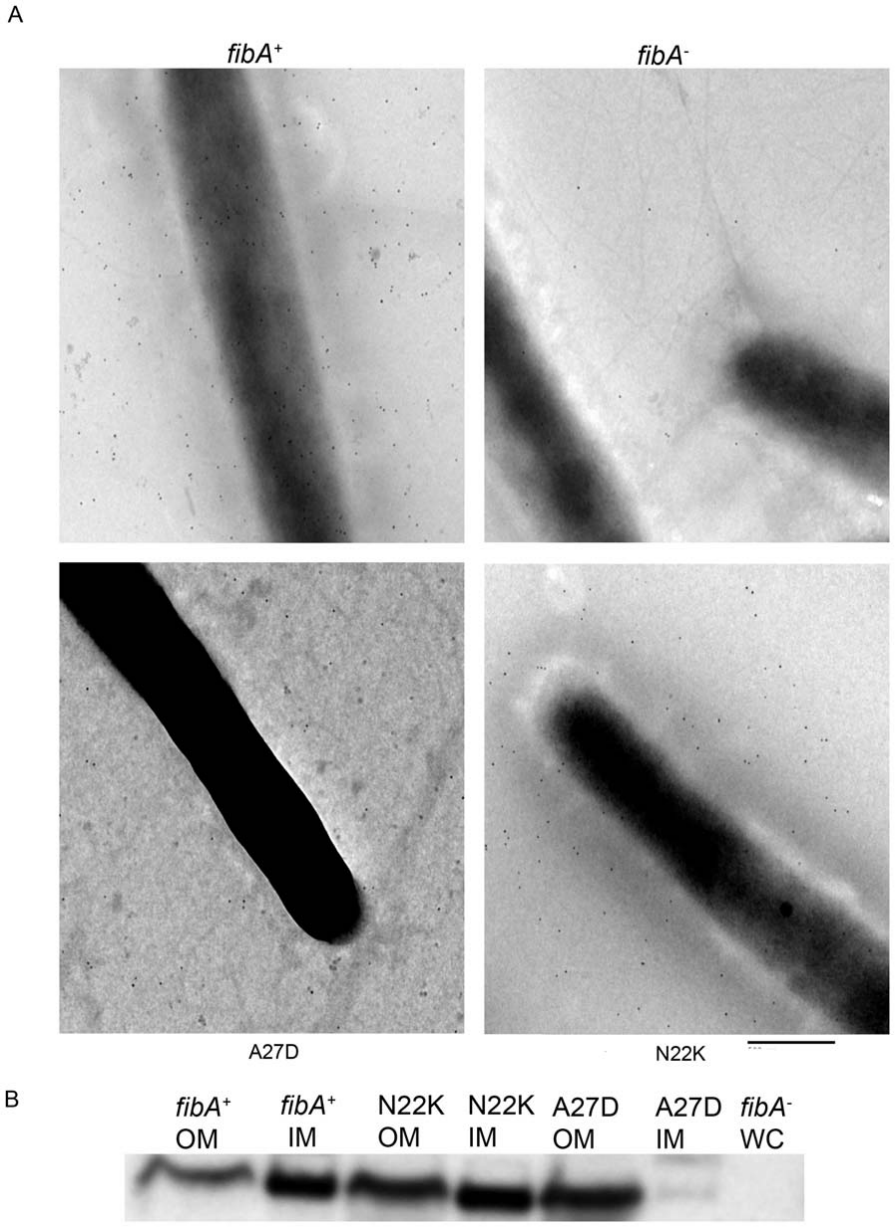
Identification of *M. xanthus* lipoprotein sorting signals. (A) Immuno transmission electron microscopy of developing cells using monoclonal antibody Mab2105. *M. xanthus* cells were allowed to form a biofilm on a formvar-carbon-coated nickel grid for 3 h. The cells were probed with Mab2105, which reacts primarily with FibA followed by anti-mouse antibodies conjugated with 10 nm colloidal gold particles. Bar is 500 nm. (B) Western blot analysis of membrane fractions purified from 7–8 h developing cells.

Approximately 70 gold particles cell^−1^ are associated with the ECM and the cell surface of strain LS2760, which produces WT FibA (Figure 4A). In contrast only a few gold particles were attached to the surfaces of LS2208 (Δ*fibA*) or LS2764 (A27D) (3.1 and 6.1 particles cell^−1^, respectively). Clearly, the +7 alanine is essential for localizing FibA to the ECM. Membrane separation of 7 h developing cells was performed in order to examine the localization of FibA to IM and OM locations. WT FibA is distributed almost equally in IM and OM during starvation suggesting that transport of FibA to the ECM occurs stepwise (Figure 4B). Conversely, the A27D change led to exclusively OM localization.

Because +2 lysine mediates retention of IM proteins (Figure 3B, [31]) we wondered whether FibA with +2 lysine would be retained in the IM. Site directed mutagenesis was used to replace an asparagine (AAC) codon with a lysine (AAG) codon at the 22^nd^ position (+2 in mature FibA). The modified *fibA* gene was also expressed in pZJY156 under control of the constitutive *pilA* promoter and introduced into LS2208. The strain produced comparable amounts of FibA to the WT (Figure 4A). Immuno transmission electron microscopy of this strain revealed large numbers of gold particles associated with the ECM (112 particles cell^−1^) suggesting that the N22K substitution does not abort ECM localization of FibA. Furthermore, N22K FibA showed similar distribution to the WT in IM and OM further confirming that lysine at the +2 position does not exclusively target FibA to the IM (Figure 4B). These results suggest that ECM lipoproteins are sorted differently than those retained in the IM.

## Discussion

A stepwise approach was used to identify OM porin proteins from the 7331 member *M. xanthus* proteome because no single program was successful. Our approach involved parsing the proteome with existing software, Signal P, Lipo P and TMHMM, to generate a smaller pool of candidates. Those proteins that possessed a type I signal sequence, but were devoid of transmembrane helices and a lipobox were examined using TMBETA-SVM and TMBETADISC-RBF. This approach identified 228 putative β-barrel proteins and dramatically reduced the number of false positives. When TMBETADISC-RBF was used on the whole *M. xanthus* proteome, it identified 915 β-barrel proteins (12% of the genome), which is far greater than is typical of Gram-negative bacteria.

Out of 228 putative β-barrel proteins, 54 were identified by LC-MS/MS in the vegetative cell OM fraction. In a complementary approach, Kahnt et al used biotinlyation of *M. xanthus* whole cells and OM vesicles to identify β-barrel proteins [9]. Kahnt et al used PRED-TMββ to identify β-barrel proteins from among the biotinylated proteins, which we found to generate over 50% false positives. Furthermore, 22/298 biotinylated proteins that were not predicted to be β-barrel proteins in the Kahnt et al study are predicted by our work to be β-barrel proteins. All in all, 43 proteins were identified by LC-MS/MS in both studies (Table 3). Because most membrane protein enrichment methods cannot avoid protein contamination from other cellular compartments, use of a robust bioinformatic approach can help accurately identify integral OM proteins from a pool of enriched candidates.

**Figure 5:**
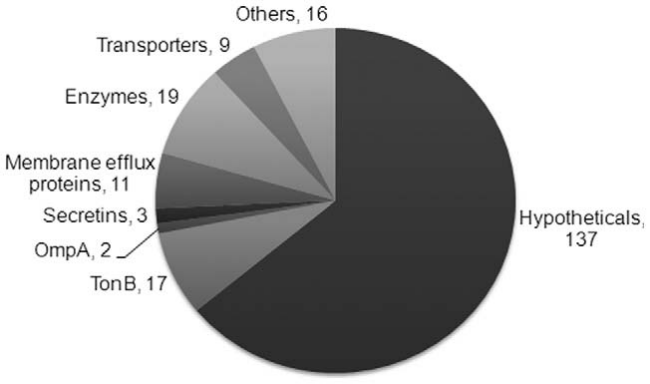
Pie chart classifying 228 OM β-barrel proteins according to function. Most *M. xanthus* OM proteins have no known function. The second major class of proteins includes TonBs, which are required for transport of a specific substrate. Transport of small molecules are carried out by transporters and OmpA, membrane efflux proteins are required for export of toxins and secretins form a large OM pore that allow export by Type II secretion system.

The majority (∼60%) of the predicted OM proteins have no known functions (Figure 5). Many putative β-barrel proteins are predicted to be involved in transport. *M. xanthus* potentially encodes 17 TonB dependent receptors of which only five were detected in vegetative cells. TonB dependent receptors are involved in energy dependent uptake of specific substrates, such as iron, which may be poorly permeable across the membrane or may be present in very low concentrations in the environment. The energy for transport is derived from the proton motive force across the IM and is delivered by a protein complex consisting of TonB, ExbB and ExbD [33], [34].

The C-signal is a developmental signal produced by the *csgA* gene. One *M. xanthus* TonB dependent receptor, Oar, appears to be essential for C-signal transmission since an *oar* mutant fails to rescue sporulation of *csgA* cells in mixture. Contact and cell alignment is essential for C-signaling [35], [36]. While the inability of *oar* cells to rescue Δ*csgA* development could arise due to improper alignment of cells caused by their abnormal shapes, another possibility is that *oar* is the OM channel used to export the C-signal. The latter possibility is suggested by the fact that *oar* is also required for the periodic movement of cells in traveling waves, sometimes referred to as ripples because of their visual similarity with ripples on the surface of water. The C-signal is the only signal known to be required for rippling. Rippling begins early in development, long before *oar* cells begin to bend. Thus it remains an intriguing possibility that *oar* is the porin for the C-signal.

Unlike *csgA* mutants, which retain their long, thin shape during their defective attempt at development, *oar* mutants become deformed in a novel manner. *oar* cells begin to bend in a central location, eventually forming circular cells whose outer membrane begins to pull away from the cell. At 24 hours, nearly 2/3 of *oar* cells are deformed. Loss of *csgA* restores normal cell morphology to *oar* cells suggesting that the morphological defect is due to accumulation of the C-signal. Curiously, the morphological problem appears to begin at the center of the cells and might suggest that C-signaling is mediated by side-to-side contact rather than polar contact as previously hypothesized [35]. While some evidence suggests that the C-signal is the CsgA protein [37], indirect evidence suggests that CsgA is an enzyme that acts on lipid molecules [38]. It is possible that the product of this enzyme reaction may destabilize the cell membrane.

The most abundant ECM lipoprotein is FibA [16], [39]. Alanine at the +7 position appears to be conserved in most ECM proteins [16]. An amino acid change from alanine to aspartate at the +7 position (A27D) in FibA leads to OM retention, suggesting that the +7 alanine is crucial for export to the ECM. In WT cells FibA is found in equal amounts in IM and OM suggesting stepwise passage through the cell envelope. If secretion occurs in a stepwise manner substitution of asparagine with lysine at the +2 position might be expected to cause FibA to accumulate in the IM, the first transit point in a temporal sequence. However, this substitution did not block export to the OM and ECM. These results suggest the *M. xanthus* secretion system for ECM proteins differs from the one for membrane proteins.

*Klebsiella oxytoca* also possesses two mechanisms for lipoprotein transport, a Lol system that moves proteins to the OM, and a type II secretion system for the export of the cell surface exposed lipoprotein PulA [40], [41]. *K. oxytoca* uses +2 Asp as a LolA avoidance tag such that proteins with +2 Asp are retained in the inner membrane while those without it move to the OM. The work presented in this paper suggests that *M. xanthus* has a similar system. However, the PulA secretion pathway requires +2 Asp to retain PulA in the IM temporarily until the type II secretion system transports it outside the cell. FibA does not have a +2 Asp so the mechanism of secretion is fundamentally different from *K. oxytoca*. Whether FibA secretion depends on a type II secretion system remains unknown. The *M. xanthus* genome predicts the presence of two type II secretion systems and future studies should reveal whether these are utilized for lipoprotein export. While, the mechanism of ECM lipoprotein trafficking remains unknown, our study provides a valuable tool to identify subcellular location of various lipoproteins based on sequence information, and a method to target specific proteins to IM, OM or ECM.

## Materials and Methods

### Bioinformatic analysis

*M. xanthus* protein sequences were obtained from NCBI (ftp://ftp.ncbi.nih.gov/genomes/Bacteria/Myxococcus_xanthus_DK_1622/NC_008095.faa). Prediction of signal peptide, lipoprotein, and transmembrane helices in protein sequences were made using Signal P (http://www.cbs.dtu.dk/services/SignalP/), Lipo P (http://www.cbs.dtu.dk/services/LipoP/) and TMHMM (http://www.cbs.dtu.dk/services/TMHMM/) respectively[10], [17], [42]. *M. xanthus* β-barrel domain proteins were obtained from the Pfam database (http://pfam.janelia.org/). The majority of the β-barrel domain proteins belong to the OM β-barrel protein (MBB) superfamily.

Six β-barrel prediction methods were evaluated for their ability to discriminate *M. xanthus* β-barrel proteins including TMB-HUNT (http://bmbpcu36.leeds.ac.uk/~andy/betaBarrel/AACompPred/aaTMB_Hunt.cgi), TMBETA-SVM (http://tmbeta-svm.cbrc.jp/), TMBETA-NET (http://psfs.cbrc.jp/tmbeta-net/), TMBETADISC-RBF (http://rbf.bioinfo.tw/~sachen/OMPpredict/TMBETADISC-RBF.php), PRED-TMBB (http://bioinformatics.biol.uoa.gr/PRED-TMBB/input.jsp) and BOMP (http://services.cbu.uib.no/tools/bomp) [42], [43], [44], [45], [46], [47].

### Bacterial strains and growth condition

Table 6 lists the bacterial strains, plasmids and primers used in this study. *M. xanthus* DK1622 cells were grown in CYE broth [1% Bacto casitone (Difco), 0.5% yeast extract (Difco), 10 mM 4-morpholinepropanesulfonic acid (MOPS) buffer (pH 7.6), and 0.1% MgSO_4_)] at 32°C with vigorous shaking. To solidify the media, Bacto agar (Difco) was added at a concentration of 1.5%. *E. coli* cells were grown in Luria-Bertani (LB) medium. Kanamycin was added to CYE or LB media at a final concentration of 50 µg ml^−1^.

**Table 6:** Bacterial strains, plasmids and primers used in this study.

*M. xanthus* strains	Genotype	Reference or source
DK1622	Wild type	[56]
LS2208	Δ*fibA*	Lawrence Shimkets
LS2441	Δ*csgA*	Lawrence Shimkets
LS2453	*oar*, Km^r^	Lawrence Shimkets
LS2456	*oar csgA*, Km^r^	Lawrence Shimkets
LS2760	LS2208 containing plasmid pSTB31, Km^r^	This study
LS2761	LS2208 containing plasmid pSTB27, Km^r^	This study
LS2764	LS2208 containing plasmid pSTB28.1, Km^r^	This study
JD300	*esg*, Km^r^	[57]
**Plasmids**		
pCR2.1-TOPO	Cloning vector	Invitrogen
pZJY156	Shuttle vector	[52]
pUC19	Cloning vector	[58]
pSTB20	pCR2.1-TOPO carrying *pilA* promoter and *fibA* gene	This study
pSTB21	pCR2.1-TOPO carrying full length *fibA*	This study
pSTB22	pUC19 carrying 500 bp, XbaI-SalI fragment from pSTB20	This study
pSTB23	pSTB22 with N22K substitution in the FibA	This study
pSTB24.1	pSTB22 with A27D substitution in the FibA	This study
pSTB25	500 bp, XbaI-SalI from pSTB23 cloned into pSTB21	This study
pSTB26.1	500 bp, XbaI-SalI from pSTB24.1 cloned into pSTB21	This study
pSTB27	pZJY156 carrying *pilA* promoter and modified *fibA* from pSTB25	This study
pSTB28.1	pZJY156 carrying *pilA* promoter and modified *fibA* from pSTB26.1	This study
pSTB31	pZJY156 carrying *pilA* promoter and WT *fibA* from pSTB20	This study
**Primers** 1		
A	5′TCTAGAGGGAGCGCTTCGGATGCGTAGGCTGATCG 3′	
B	5′CTTCTGCACGAGCATGGGGGTCCTCAGAGAAGGTTGCAACGG 3′	
C	5′ ACCCCCATGCTCGTGCAGAAGAGAGTTCGCGGAGCG 3′	
D	5′ GGTACCCCTCGAGCCGCTGCCCAAGTAG 3′	
FibA2DF	5′ GAGTCCACCCCTGACCCCGAGGCCGAC 3′	
FibA2DR	5′ GTCGGCCTCGGGGTCAGGGGTGGACTC3′	
FibAKF	5′ GTTGTCGGTTGCAAGGAGTCCACCCCTGCC 3′	
FibAKR	5′ GGCAGGGGTGGACTCCTTGCAACCGACAAC 3′	

1 Underline indicates an overlap of 21 nucleotides.

### Membrane separation

Membrane separation was carried out as described by Simunovic et al with a few modifications [48]. A 1 L culture of *M. xanthus* DK1622 cells was grown to a density of 2×10^8^ cells ml^−1^. Cells were harvested by centrifugation, washed with chilled distilled water, and resuspended in 40 to 50 ml of 23.5% sucrose in 20 mM N-2-hydroxyethylpiperazine-N′-2-ethanesulfonic acid (HEPES), pH 7.6. Freshly prepared chicken egg white lysozyme (300 µg ml^−1^) (Sigma Chemical Co., St. Louis, Mo.) and EDTA (pH 7.6) (1 mM) were added, and incubated overnight at 4°C with gentle stirring. The cells were resuspended in 6 ml of ice-cold double-distilled water with vigorous pipetting to induce spheroplast formation, and stirred for 30 min at 4°C. Spheroplasts were collected by centrifugation at 12,000× *g* for 10 min at 4°C. Following centrifugation the supernatant was collected and saved while the pellet was resuspended in 3 volumes of 5 mM EDTA (pH 7.6). One tablet of complete EDTA-free protease inhibitor (Roche, Indianapolis, Ind.) was added, and the suspension stirred for 1 h. The supernatant was added back to the suspension and stirred for an additional 30 min. 1 ml of RNase A (10 mg ml^−1^, Sigma Chemical Co.) and 1 ml of DNase type II (10 mg ml^−1^, Sigma Chemical Co.) were added, and stirred for 30 min. Rod-shaped cells were pelleted by centrifugation at 5000× g for 10 min at 4°C. Spheroplasts were collected by ultracentrifugation at 100,000× *g* for 3 h at 4°C in a 70.1 Ti rotor (Beckman Coulter). Membrane pellets were resuspended in 23.5% sucrose in 20 mM HEPES, 5 mM EDTA, pH 7.6 using a Dual 21 tissue homogenizer (Kimble Kontes, Vineland, N.J.), and incubated overnight with gentle stirring at 4°C. The membrane suspension was loaded on top of a three-step gradient consisting of 10 ml of 60% sucrose, 10 ml of 48% sucrose, 10 ml of 35% sucrose in 20 mM HEPES, 5 mM EDTA, pH 7.6. The membrane fractions were separated by ultracentrifugation at 120,000× *g* for 4 h at 4°C in a Beckman swinging bucket rotor (SW28). The OM fraction migrated to the middle of the 35% sucrose layer. It was collected, diluted with HE0.1 buffer [20 mM HEPES, 0.1 mM EDTA, pH 7.6], concentrated by ultracentrifugation at 120,000× *g* for 3 h at 4°C in a 70.1 Ti rotor, then stored at −20°C.

The membrane enrichments were collected as described by Simunovic et al [23]. The membrane enrichment that migrated at the 48/60% sucrose layer interface consisted of IM and hybrid membrane (HM) fraction, which were collected and concentrated by ultracentrifugation using a SW28 rotor at 120,000× *g* for 4 h at 4°C. Concentrated enrichments were layered on top of a discontinuous sucrose gradient consisting of 4 ml 70% sucrose, 4 ml 60% sucrose, 15 ml 55% sucrose, 3 ml 40% sucrose, and 3 ml 30% sucrose in 20 mM HEPES, 5 mM EDTA, pH 7.6. The gradients were centrifuged using SW28 rotor at 70,000× *g* for 20 h. The membrane enrichment that migrated between the 60 and 70% sucrose layers consisted of IM fraction. This fraction was collected, concentrated by ultracentrifugation, then stored at −20°C for proteomic analysis.

Membrane separation of developing cells was carried out by growing a 500 ml culture to a final density of 5×10^8^ cell ml^−1^. The cells were harvested by centrifugation and resuspended in 10 ml water. The cells were spread on two 33- by 22-cm trays containing TPM agar [10 mM Tris HCl, pH 7.6, 1 mM KH(H_2_)PO_4_, pH 7.6, 10 mM MgSO_4_, 1.5% agar (Difco)] and incubated at 32°C for 7 h. Developing cells were harvested with a razor blade and resuspended in 40 to 50 ml of 23.5% sucrose in 20 mM HEPES, pH 7.6. The membrane separation was carried out as described above.

### Phenol extraction of OM proteins

Phenol extraction of OM proteins was carried out as described in Hancock and Nikaido [49]. An equal volume of 88% phenol, pH 6.8, was added to the OM protein sample that was prepared and frozen as mentioned above. The OM sample was incubated at 70°C for 10 min. The mixture was immediately cooled on ice for 10 min, and then centrifuged for 10 min at 5000× *g*. The upper, aqueous layer was discarded. To the interface and the phenol phase an equal volume of distilled water was added, incubated at 70°C for 10 min, cooled on ice, and centrifuged at 5000× *g* for 10 min. After the aqueous phase was discarded, protein was extracted from the phenol phase using two volumes of acetone each time. The acetone fractions were combined and the acetone was removed by air-drying. The pellet was resuspended in 100 µl of 1× PBS buffer [137 mM NaCl, 2.7 mM KCl, 4.3 mM Na_2_HPO_4_, 1.47 mM KH_2_PO_4_, pH 7.4].

### In-gel trypsin digestion

150 µg protein was boiled in loading buffer [52.5 mM Tris-HCl, pH 6.8, 2% SDS, 25% glycerol, 0.01% bromophenol blue, 100 mM dithiothreitol (DTT)] for 10 min and cooled on ice for 10 min. The sample was loaded on a 4–20% gradient polyacrylamide gel. The gel was run at 70 V until the dye entered the gel. Protein detection was performed using Bio-safe Coomassie Stain (Bio-Rad). The portion of the gel containing protein was cut into small pieces, and destained with 100 µl of water for 15 min. The gel pieces were washed sequentially for 15 minutes each with 50% acetonitrile, 100% acetonitrile, and 100 mM ammonium bicarbonate containing 50% acetonitrile (vol/vol). The gel pieces were dried under vacuum, treated with 100 µl of 10 mM DTT in 40 mM ammonium bicarbonate at 56°C for 45 min, alkylated with 100 µl of 55 mM iodoacetamide, 40 mM ammonium bicarbonate, and incubated for 30 min at room temperature in the dark. The gel pieces were washed with acetonitrile for 15 min, and then dried under vacuum. The gel pieces were rehydrated with 2 µg µl^−1^ proteomics-grade trypsin (Promega) in 40 mM ammonium bicarbonate and incubated at 37°C overnight. Solutions from multiple trypsin digestions were pooled. The gel slices were washed once with 50% acetonitrile in 25 mM ammonium bicarbonate, twice with 5% formic acid, and twice with acetonitrile for 15 min each. The washes were combined with the solutions from the previous step and dried under vacuum.

### Identification of OM proteins by LC/MS-MS

The peptides obtained from trypsin digestion were loaded on a PicoFrit C_18_ column (8-cm by 50-µm) (New Objective, Woburn, MA), and separated on an Agilent 1100 capillary LC (Palo Alto, CA), which interfaced directly to a LTQ linear ion trap mass spectrometer (Thermo Electron, San Jose, CA). The mobile phases consisted of A (H_2_O and 0.1% formic acid) and B (acetonitrile and 0.1% formic acid). The peptides were eluted from the column during a 90 min linear gradient from 5 to 60% of total solution composed of mobile phase B into the mass spectrometer at a flow rate of 200 ηl min^−1^. MS/MS spectra were acquired on the nine most abundant precursor ions from each MS scan with a repeat count and duration of 1 and 5 s each. Dynamic exclusion was enabled for 200 s. The MS/MS spectra were converted into peak lists by mzMXL2Other and ReAdW software [50].

Database searches were performed using Mascot 1.9 software (Matrix Science, Boston, MA) against a *M. xanthus* protein database obtained from NCBI. The search parameters included full tryptic enzymatic cleavage, up to three missed cleavages, peptide tolerance of 1000 ppm, fragment ion tolerance of 0.6 Da. Fixed modification was set as carbamidomethyl due to carboxyamidomethylation of cysteine residues (+57 Da) while variable modification was set as oxidation of methionine residues (+16 Da) and deamidation of asparagines residues (+1 Da). The proteins identified were statistically validated using ProValT algorithm as implemented in ProteoIQ (BioInquire, Athens, GA) [51]. Only proteins with a false-discovery rate of less than 1% were considered to be statistically significant.

### Extracellular complementation

*M. xanthus* cells were grown to a cell density of 5×10^8^ cells ml^−1^, harvested and resuspended to a final concentration of 5×10^9^ cells ml^−1^. *oar* cells were mixed with various strains in 1∶1 ratio and 10 µl of the cell mixture was spotted on TPM agar plates. The plates were incubated at 32°C and after five days digital images were acquired.

### Microscopic analysis

5×10^8^ *M. xanthus* cells were spotted on TPM agar plates and incubated for 24 h. Cells were resuspended in a drop of TPMF buffer [TPM containing 10% ficoll], and examined with a phase contrast microscope (Leica Microsystems, DM55008). Digital images were obtained at 1000× magnification using a QIQICAM FAST 1394 camera (Compix Inc).

### Site directed mutagenesis and cloning of *fibA*

The *fibA* gene was expressed from pZJY156 [52]. The *fibA* gene was fused with the *pilA* promoter using the gene splicing by overlap extension (SOEing PCR) method [53]. The primers are listed in Table 6. Primers A and B were used for amplification of the *pilA* promoter including the ribosomal binding site and the start codon [54]. Primers C and D were used for the amplification of full length *fibA*. Primers B and C were designed to have an overlap of 21 nucleotides (underlined region). *M. xanthus* DK1622 genomic DNA was used as a template. The two PCR products and primer pair A and D were then used for SOEing PCR. PCR products were separated on 0.8% agarose, excised, extracted using the Gel Extraction kit (Qiagen), and cloned into pCR2.1-TOPO (Invitrogen) to create pSTB20. Full length *fibA* was cloned into pCR2.1-TOPO to create pSTB21. A 500 bp, XbaI-SalI fragment containing the *pilA* promoter and the N-terminal region of FibA from pSTB20 was cloned into pUC19 to create pSTB22, which was used as the template for site directed mutagenesis. The primers used for the site directed mutagenesis are listed in Table 6. Primers FibA2DF and FibA2DR were used for replacing alanine with aspartate at 27^th^ position while FibAKF and FibAKR were used for replacing asparagine with lysine at the 22^nd^ position (Table 6). PCR was carried out using a high fidelity DNA polymerase I (Expand High Fidelity PCR system, Roche). The PCR products obtained were treated with DpnI to eliminate the methylated template, and then transformed into *E. coli* Top10 cells. Plasmids were isolated from transformants and sequenced. The plasmids encoding FibA with N22K or A27D amino acid substitutions were called pSTB23 and pSTB24.1 respectively. The 500 bp, XbaI-SalI from pSTB23 and pSTB24.1 were cloned at the same site in pSTB21 to create pSTB25 and pSTB26.1, respectively. The new plasmids contained full length *fibA* encoding the N22K or A27D substitutions expressed from the *pilA* promoter. The plasmids, pSTB25 and pSTB26.1 were digested with XbaI and EcoRI, and the 2800 bp fragment containing the *pilA* promoter and *fibA* gene was cloned into pZJY156 to create pSTB27 and pSTB28.1 plasmids. These plasmids were then transformed into *M. xanthus* LS2208 to create LS2761 and LS2764. Full length *fibA* expressed from the *pilA* promoter was cloned into pZJY156 to create pSTB31 and also transformed into *M. xanthus* LS2208 to create LS2760. Expression of *fibA* from the *pilA* promoter was verified by western blotting using monoclonal antibody Mab2105 [55].

### Western blot analysis

5 µg of cell lysate or 10 µg of membrane fractions, were separated on a 4–20% SDS-PAGE gradient gel (Bio Rad). Proteins were transferred to an Immobilin-P, PVDF membrane (Milipore). The membrane was blocked with 3% bovine serum albumin (BSA) in PBST (1× PBS containing 0.1% Tween 20). The proteins were probed with the Mab2105 (1∶500 dilution) or anti-CsgA (1∶5000) that was prepared in PBST containing 0.1% BSA [55]. This was followed by washing three times with PBST. The membrane blot was then probed with horseradish peroxidase-conjugated goat anti-mouse IgG or anti-rabbit IgG, which were diluted to 1∶10,000 in PBST containing 0.1% BSA. The membrane was washed three times with PBST and developed with the ECL luminescence detection kit (Amersham).

### Electron microscopy

*M. xanthus* cells were grown to a density of 5×10^8^ cells ml^−1^ and diluted to 3.3×10^6^ cells ml^−1^in CYE or CYEK (CYE containing 50 µg ml^−1^ kanamycin) broth. 4 ml of the cell suspension and a formvar-carbon-coated nickel grid (Electron Microscope Sciences) was transferred to a petri plate (60×15 mm). The plate was incubated at 32°C for 12 h. A thin biofilm on the surface of the grid was allowed to form. Starvation was induced by replacing the CYE or CYEK broth with cohesion buffer [10 mM MOPS, pH 6.8, 1 mM MgCl_2_, 1 mM CaCl_2_] and incubated for 3 h at 32°C. The grid was treated with 2% glutaraldehyde for 15 min at room temperature followed by washing five times with the cohesion buffer. The grid was blocked with 5% bovine serum albumin (BSA) in cohesion buffer for 45 min at room temperature. The grid was treated with Mab2105 antibody (1∶20 dilution) prepared in cohesion buffer containing 5% BSA for 45 min at room temperature followed by washing three times with cohesion buffer [55]. The grid was then treated with anti-mouse antibody (1∶100 dilutions) conjugated to 10 nm colloidal gold particles (Sigma-Aldrich), and incubated for 30 min at room temperature. The grid was washed three times with the cohesion buffer. The grid was washed three times with water, allowed to air dry, and observed under a FEI Technal transmission electron microscope operated at 200 kV.

## Supporting Information

Table S1 228 OM proteins identified by bothTMBETA-SVM and TMBETADISC-RBF.
(DOC)

Table S2 List of non-outer membrane integral proteins identified by LC-MS/MS.
(DOC)
