# Supplementary material for: Identification and Localization of Myxococcus xanthus Porins and Lipoproteins
Source: PLoS One. 2011 Nov 22;6(11):e27475. doi: 10.1371/journal.pone.0027475 (PMC3222651; doi:10.1371/journal.pone.0027475)
Supplement: Table S1 — 228 OM proteins identified by bothTMBETA-SVM and TMBETADISC-RBF. (DOC) [file pone.0027475.s001.doc]

**Supplementary table S1.** 228 OM proteins identified by bothTMBETA-SVM and TMBETADISC-RBF.

| **MXAN** | **Gene name** | **Protein function** |
| --- | --- | --- |
| MXAN0021 |  | Hypothetical protein |
| MXAN0033 |  | Hypothetical protein |
| MXAN0063 |  | Hypothetical protein |
| MXAN0129 |  | Hypothetical protein |
| MXAN0162 |  | Hypothetical protein |
| MXAN0182 |  | PQQ enzyme repeat domain protein |
| MXAN0219 |  | Hypothetical protein |
| MXAN0224 |  | Hypothetical protein |
| MXAN0251 |  | Mce family protein |
| MXAN0271 |  | Hypothetical protein |
| MXAN0272 |  | TonB-dependent receptor |
| MXAN0355 |  | Putative pilus biogenesis operon protein |
| MXAN0391 |  | Oxidoreductase, aldo/keto reductase family |
| MXAN0413 |  | Hypothetical protein |
| MXAN0500 |  | Hypothetical protein |
| MXAN0518 |  | TonB-dependent receptor |
| MXAN0538 |  | Hypothetical protein |
| MXAN0551 |  | Hypothetical protein |
| MXAN0562 |  | Phosphate-selective porin O and P |
| MXAN0585 |  | Hypothetical protein |
| MXAN0589 |  | Hypothetical protein |
| MXAN0653 |  | Peptidase, S8A (subtilisin) subfamily |
| MXAN0662 |  | Hypothetical protein |
| MXAN0679 |  | Hypothetical protein |
| MXAN0751 |  | Amino acid/amide ABC transporter substrate-binding protein |
| MXAN0774 |  | Oxidoreductase, short chain dehydrogenase/reductase family |
| MXAN0794 |  | Hypothetical protein |
| MXAN0821 |  | TonB dependent receptor |
| MXAN0855 |  | Putative chemotaxis motb protein |
| MXAN0856 |  | TonB dependent receptor |
| MXAN0924 |  | Hypothetical protein |
| MXAN0940 |  | Hypothetical protein |
| MXAN0945 |  | Tetratricopeptide repeat protein |
| MXAN0959 | *sbcC* | Nuclease sbccd, C subunit |
| MXAN0990 |  | Cation efflux system protein cusc |
| MXAN1003 |  | Hypothetical protein |
| MXAN1020 |  | Hypothetical protein |
| MXAN1021 |  | Hypothetical protein |
| MXAN1051 |  | Hypothetical protein |
| MXAN1070 | *dacB* | D-alanyl-D-alanine carboxypeptidase/D-alanyl-D-alanine-endopeptidase |
| MXAN1147 |  | Hypothetical protein |
| MXAN1183 |  | Spermine/spermidine synthase family protein |
| MXAN1226 |  | Fibronectin type III domain protein |
| MXAN1242 |  | Hypothetical protein |
| MXAN1263 |  | Hypothetical protein |
| MXAN1316 |  | TonB dependent receptor |
| MXAN1329 |  | Hypothetical protein |
| MXAN1368 |  | Hypothetical protein |
| MXAN1369 |  | Prepilin-type N-terminal cleavage/methylation domain protein |
| MXAN1426 |  | Hypothetical protein |
| MXAN1559 |  | Hypothetical protein |
| MXAN1608 |  | Hypothetical protein |
| MXAN1688 |  | TonB dependent receptor |
| MXAN1700 |  | Tetratricopeptide repeat protein |
| MXAN1897 |  | Hypothetical protein |
| MXAN1904 |  | Hypothetical protein |
| MXAN1916 |  | Hypothetical protein |
| MXAN1923 |  | Hypothetical protein |
| MXAN1948 |  | Tetratricopeptide repeat protein |
| MXAN1967 |  | Putative peptidase, S8 (subtilisin) family |
| MXAN1999 |  | Hypothetical protein |
| MXAN2203 |  | Hypothetical protein |
| MXAN2277 |  | Hypothetical protein |
| MXAN2374 |  | Hypothetical protein |
| MXAN2380 |  | Hypothetical protein |
| MXAN2412 |  | Hypothetical protein |
| MXAN2417 |  | Hypothetical protein |
| MXAN2422 |  | Hypothetical protein |
| MXAN2426 |  | Hypothetical protein |
| MXAN2466 |  | Hypothetical protein |
| MXAN2481 |  | Hypothetical protein |
| MXAN2508 |  | Putative prepilin-type N-terminal cleavage/methylation domain protein |
| MXAN2514 | *gspD* | General secretion pathway protein D |
| MXAN2515 | *gspC* | General secretion pathway protein C |
| MXAN2520 |  | MORN variant repeat protein |
| MXAN2537 |  | TldD/PmbA family protein |
| MXAN2539 |  | Hypothetical protein |
| MXAN2552 |  | Tetratricopeptide repeat protein |
| MXAN2561 |  | Prepilin-type N-terminal cleavage/methylation domain protein |
| MXAN2562 |  | Hypothetical protein |
| MXAN2584 |  | Hypothetical protein |
| MXAN2619 |  | Tetratricopeptide repeat protein |
| MXAN2659 |  | Hypothetical protein |
| MXAN2690 |  | AsmA family protein |
| MXAN2782 |  | Hypothetical protein |
| MXAN2850 |  | Hypothetical protein |
| MXAN2864 |  | Hypothetical protein |
| MXAN2915 |  | Putative adventurous gliding motility protein |
| MXAN2952 |  | Hypothetical protein |
| MXAN2995 |  | Peptidase, S1C (protease Do) subfamily |
| MXAN3106 |  | Protein transporter, outer bacterial membrane secretin (secretin) family |
| MXAN3129 |  | Prolyl oligopeptidase, serine peptidase |
| MXAN3165 |  | Hypothetical protein |
| MXAN3242 |  | Hypothetical protein |
| MXAN3373 |  | Hypothetical protein |
| MXAN3424 |  | Outer membrane efflux protein |
| MXAN3482 |  | Hypothetical protein |
| MXAN3483 |  | Hypothetical protein |
| MXAN3553 |  | Hypothetical protein |
| MXAN3564 |  | Peptidase, M36 (fungalysin) family |
| MXAN3676 |  | Hypothetical protein |
| MXAN3719 |  | Fibronectin type III domain protein |
| MXAN3723 |  | Hypothetical protein |
| MXAN3729 |  | PQQ enzyme repeat domain protein |
| MXAN3745 |  | Hypothetical protein |
| MXAN3768 |  | Mce family protein |
| MXAN3774 |  | Hypothetical protein |
| MXAN3774 |  | Dipeptidyl-peptidase 7, serine peptidase |
| MXAN3780 |  | Patatin-like phospholipase family protein |
| MXAN3814 |  | Hypothetical protein |
| MXAN3824 | *gspG* | General secretion pathway protein G |
| MXAN3883 |  | Protein transporter, outer membrane fimbrial usher porin (FUP) family |
| MXAN3905 |  | Outer membrane efflux protein |
| MXAN3953 |  | Hypothetical protein |
| MXAN4014 |  | Von Willebrand factor type A domain protein |
| MXAN4092 |  | Hypothetical protein |
| MXAN4109 |  | Hypothetical protein |
| MXAN4115 |  | Transposase orf3, IS66 family |
| MXAN4153 |  | Hypothetical protein |
| MXAN4175 |  | Efflux transporter, RND family, MFP subunit |
| MXAN4176 |  | Outer membrane efflux protein |
| MXAN4181 |  | Hypothetical protein |
| MXAN4198 |  | Putative outer membrane macrolide efflux protein |
| MXAN4200 |  | Putative macrolide-specific efflux protein |
| MXAN4293 |  | Hypothetical protein |
| MXAN4308 |  | Hypothetical protein |
| MXAN4365 |  | Transporter, outer membrane receptor (OMR) family |
| MXAN4381 |  | Hypothetical protein |
| MXAN4382 |  | Hypothetical protein |
| MXAN4390 |  | BNR/Asp-box repeat domain protein |
| MXAN4407 |  | Hypothetical protein |
| MXAN4559 |  | TonB dependent receptor |
| MXAN4561 |  | Tetratricopeptide repeat protein |
| MXAN4652 |  | Putative Flp pilus assembly protein CpaB |
| MXAN4658 |  | Pilus biogenesis protein, TadE family |
| MXAN4695 |  | Spo IID/lytB domain protein |
| MXAN4728 | *omp85* | Outer membrane protein, OMP85 family |
| MXAN4746 |  | TonB dependent receptor |
| MXAN4821 |  | Sulfate ABC transporter, periplasmic sulfate-binding protein |
| MXAN4837 | *celA* | Endoglucanase |
| MXAN4859 |  | Ankyrin repeat protein |
| MXAN4876 |  | Hypothetical protein |
| MXAN4897 |  | Hypothetical protein |
| MXAN4911 |  | Hypothetical protein |
| MXAN5013 |  | Hypothetical protein |
| MXAN5023 |  | TonB dependent receptor |
| MXAN5030 |  | Efflux transporter, HAE1 family, outer membrane efflux protein |
| MXAN5117 |  | Hypothetical protein |
| MXAN5191 |  | Hypothetical protein |
| MXAN5200 |  | Hypothetical protein |
| MXAN5291 | *mtgA* | Monofunctional biosynthetic peptidoglycan transglycosylase |
| MXAN5292 |  | Hypothetical protein |
| MXAN5299 |  | Peptidase, S9C (acylaminoacyl-peptidase) subfamily |
| MXAN5304 |  | Hypothetical protein |
| MXAN5326 |  | Putative phytase |
| MXAN5375 |  | Hypothetical protein |
| MXAN5391 |  | Hypothetical protein |
| MXAN5442 |  | Thermolysin, metallo peptidase |
| MXAN5453 |  | Hypothetical protein |
| MXAN5454 |  | Peptidase, M36 (fungalysin) family |
| MXAN5491 |  | Hypothetical protein |
| MXAN5522 |  | Lactonizing lipase |
| MXAN5685 |  | Hypothetical protein |
| MXAN5693 |  | DNA/RNA non-specific endonuclease |
| MXAN5720 |  | Metallo-beta-lactamase family protein |
| MXAN5743 |  | Hypothetical protein |
| MXAN5756 | *tolB* | TolB protein |
| MXAN5772 | *pilQ* | Type IV pilus secretin PilQ |
| MXAN5809 |  | Hypothetical protein |
| MXAN5819 |  | Hypothetical protein |
| MXAN5822 |  | Hypothetical protein |
| MXAN5835 |  | Hypothetical protein |
| MXAN5869 |  | Hypothetical protein |
| MXAN5875 |  | Hypothetical protein |
| MXAN5931 |  | Hypothetical protein |
| MXAN5970 |  | Peptidase, S8 (subtilisin) family |
| MXAN5998 |  | Putative lipase |
| MXAN6040 |  | Hypothetical protein |
| MXAN6044 |  | TonB-dependent receptor |
| MXAN6090 |  | Hypothetical protein |
| MXAN6196 |  | Hypothetical protein |
| MXAN6236 |  | Putative polysaccharide-degrading enzyme |
| MXAN6246 |  | OmpA domain protein |
| MXAN6272 |  | Hypothetical protein |
| MXAN6333 |  | Hypothetical protein |
| MXAN6404 |  | Hypothetical protein |
| MXAN6405 |  | Hypothetical protein |
| MXAN6409 |  | Putative beta-lactamase |
| MXAN6429 |  | Hypothetical protein |
| MXAN6465 |  | Hypothetical protein |
| MXAN6487 |  | Outer membrane efflux protein domain protein |
| MXAN6547 |  | TonB-dependent receptor |
| MXAN6579 |  | TonB-dependent receptor |
| MXAN6674 |  | Hypothetical protein |
| MXAN6679 |  | Hypothetical protein |
| MXAN6703 |  | Gliding motility protein |
| MXAN6716 |  | TonB dependent receptor |
| MXAN6737 |  | TonB-dependent receptor |
| MXAN6767 |  | Hypothetical protein |
| MXAN6774 |  | Hypothetical protein |
| MXAN6845 |  | TonB-dependent receptor |
| MXAN6911 |  | TonB-dependent receptor |
| MXAN7034 |  | Hypothetical protein |
| MXAN7037 |  | MotB chemotaxis proteins |
| MXAN7039 |  | Hypothetical protein |
| MXAN7040 | *oar* | TonB dependent receptor |
| MXAN7112 |  | Hypothetical protein |
| MXAN7210 |  | Hypothetical protein |
| MXAN7331 |  | TonB-dependent receptor |
| MXAN7407 |  | Hypothetical protein |
| MXAN7436 |  | Outer membrane efflux protein |
| MXAN7464 |  | Hypothetical protein |
| MXAN7497 |  | Peptidase, M16B (mitochondrial processing peptidase beta-subunit) family |
| MXAN7513 |  | Hypothetical protein |
