# Supplementary material for: Identification and Localization of Myxococcus xanthus Porins and Lipoproteins
Source: PLoS One. 2011 Nov 22;6(11):e27475. doi: 10.1371/journal.pone.0027475 (PMC3222651; doi:10.1371/journal.pone.0027475)
Supplement: Table S2 — List of non-outer membrane integral proteins identified by LC-MS/MS. (DOC) [file pone.0027475.s002.doc]

**Supplementary table S2.** List of non-outer membrane integral proteins identified by LC-MS/MS

| **MXAN** | **Function** | **No. of peptides** |
| --- | --- | --- |
| MXAN0403 | ATP synthase F0, C subunit | 1 |
| MXAN0791 | Peptidase, M16 (pitrilysin) family | 1 |
| MXAN0870 | conserved hypothetical protein | 8 |
| MXAN0875 | Hypothetical protein | 1 |
| MXAN0976 | Putative lipoprotein | 3 |
| MXAN1080 | NADH dehydrogenase I, N subunit | 1 |
| MXAN1111 | Propionyl-CoA carboxylase, alpha subunit | 3 |
| MXAN1389 | Putative alkaline phosphatase | 13 |
| MXAN1448 | Transporter, MotA/TolQ/Exbb proton channel family | 4 |
| MXAN1449 | Putative TonB protein | 1 |
| MXAN1564 | Alkyl hydroperoxide reductase C | 5 |
| MXAN1671 | V-type H(+)-translocating pyrophosphatase | 1 |
| MXAN1994 | Ribosomal protein S9 | 1 |
| MXAN2016 | Prolyl endopeptidase precursor Pep | 12 |
| MXAN2666 | Pyruvate dehydrogenase complex | 10 |
| MXAN2667 | Pyruvate dehydrogenase complex, E1 component, pyruvate dehydrogenase, beta subunit | 11 |
| MXAN2668 | Pyruvate dehydrogenase complex , E2 component, dihydrolipoamide acetyltransferase | 7 |
| MXAN2719 | Hypothetical protein | 1 |
| MXAN2720 | Hypothetical protein | 1 |
| MXAN2728 | Putative NADH dehydrogenase I, G subunit | 1 |
| MXAN2787 | Homogentisate 1,2-dioxygenase | 2 |
| MXAN2814 | Putative N-acetylmuramoyl-L-alanine amidase | 1 |
| MXAN2967 | RND transporter, hydrophobe/amphiphile efflux-1 (HAE1) family | 3 |
| MXAN3206 | DSBA-like thioredoxin domain protein | 15 |
| MXAN3296 | Ribosomal protein S7 | 4 |
| MXAN3305 | Ribosomal protein S3 | 1 |
| MXAN3309 | Ribosomal protein L14 | 1 |
| MXAN3311 | Ribosomal protein L5 | 1 |
| MXAN3316 | Ribosomal protein S5 | 5 |
| MXAN3323 | Ribosomal protein S13 | 4 |
| MXAN3326 | DNA-directed RNA polymerase, alpha subunit | 1 |
| MXAN3327 | Ribosomal protein L17 | 4 |
| MXAN3370 | Ribosomal protein L3 | 3 |
| MXAN3539 | Succinate dehydrogenase, flavoprotein subunit | 4 |
| MXAN3549 | Ribosomal protein L19 | 6 |
| MXAN3549 | Ribosomal protein L19 | 6 |
| MXAN3556 | Hypothetical protein | 3 |
| MXAN3743 | Hypothetical protein | 1 |
| MXAN3779 | Non-ribosomal peptide synthetase/polyketide synthase | 5 |
| MXAN4217 | Alpha keto acid dehydrogenase complex, E2 component | 13 |
| MXAN4219 | Alpha keto acid dehydrogenase complex, E3 component | 18 |
| MXAN4289 | Hypothetical protein | 4 |
| MXAN4293 | Hypothetical protein | 4 |
| MXAN4299 | Non-ribosomal peptide synthase/polyketide synthase | 1 |
| MXAN4327 | Glu/Leu/Phe/Val dehydrogenase family protein | 3 |
| MXAN4467 | Chaperonin GroEL | 36 |
| MXAN4564 | 2-oxoisovalerate dehydrogenase complex, E1 component, alpha subunit | 15 |
| MXAN4565 | 2-oxoisovalerate dehydrogenase complex, E1 component, beta subunit | 15 |
| MXAN4650 | Type II secretion system protein F domain protein | 1 |
| MXAN4690 | Protein-export membrane protein SecF | 4 |
| MXAN4691 | Protein-export membrane protein SecD | 1 |
| MXAN4692 | Preprotein translocase, YajC subunit | 1 |
| MXAN4808 | Conserved hypothetical protein | 5 |
| MXAN4860 | hypothetical protein MXAN_4860 | 10 |
| MXAN4895 | Chaperonin GroEL | 33 |
| MXAN5143 | Tol-pal system protein YbgF | 3 |
| MXAN5152 | OmpA family protein | 3 |
| MXAN5344 | Ribosomal protein S2 | 3 |
| MXAN5743 | Hypothetical protein | 11 |
| MXAN6000 | Iron compound ABC transporter, periplasmic iron compound-binding protein | 1 |
| MXAN6035 | 2-oxoglutarate dehydrogenase, E1 component | 18 |
| MXAN6036 | 2-oxoglutarate dehydrogenase, E2 component, dihydrolipoamide succinyltransferase | 13 |
| MXAN6247 | Terpene synthase, metal binding domain protein | 5 |
| MXAN6248 | Cyclic nucleotide-binding domain protein | 20 |
| MXAN6249 | Cyclic nucleotide-binding domain protein | 14 |
| MXAN6483 | MotA/TolQ/Exbb proton channel family protein | 3 |
| MXAN6574 | Putative lipoprotein | 6 |
| MXAN6601 | Peptidase, S9C (acylaminoacyl-peptidase) subfamily | 3 |
| MXAN6601 | Peptidase, S9C (acylaminoacyl-peptidase) subfamily | 3 |
| MXAN6665 | Putative branched chain amino acid ABC transporter, periplasmic amino acid-binding protein | 2 |
| MXAN6805 | Ribosomal protein S4 | 2 |
| MXAN6829 | Putative late embryogenesis abundant-like protein | 4 |
| MXAN6862 | MotA/TolQ/Exbb proton channel family protein | 1 |
| MXAN6884 | Hypothetical protein | 3 |
| MXAN6913 | Cytochrome d ubiquinol oxidase, subunit I | 1 |
| MXAN6976 | Hypothetical protein | 9 |
| MXAN7028 | ATP synthase F1, alpha subunit | 4 |
| MXAN7104 | Peptidase, M3 (thimet oligopeptidase) family | 10 |
| MXAN7340 | Hypothetical protein | 3 |
| MXAN7437 | Heavy metal efflux pump, CzcA family | 1 |
